# The neurotoxin 1-methyl-4-phenylpyridinium (MPP^+^) alters hippocampal excitatory synaptic transmission by modulation of the GABAergic system

**DOI:** 10.3389/fncel.2015.00299

**Published:** 2015-08-04

**Authors:** YuYing Huang, JunFang Chen, Ying Chen, YingHan Zhuang, Mu Sun, Thomas Behnisch

**Affiliations:** ^1^Institutes of Brain Science, State Key Laboratory of Medical Neurobiology, and Collaborative Innovation Center for Brain Science, Fudan UniversityShanghai, China; ^2^Neurodegeneration Discovery Performance Unit, GSK R&DShanghai, China

**Keywords:** Parkinson’s disease, dopaminergic system, extrasynaptic GABAergic receptors, synaptic transmission, tonic inhibition, limbic system

## Abstract

The neurotoxin 1-methyl-4-phenyl-1,2,3,6-tetrahydropyridine (MPTP) induces Parkinson’s disease-like symptoms following administration to mice, monkeys, and humans. A common view is that MPTP is metabolized to 1-methyl-4-phenylpyridinium ion (MPP^+^) to induce its neurodegenerative effects on dopaminergic neurons in the substantia nigra (SN). Moreover, the hippocampus contains dopaminergic fibers, which are projecting from the ventral tegmental area, SN and pars compacta and contain the whole machinery required for dopamine synthesis making them sensitive to MPTP and MPP^+^. Here, we present data showing that acute bath-application of MPP^+^ elicited a dose-dependent facilitation followed by a depression of synaptic transmission of hippocampal Schaffer collaterals-CA1 synapses in mice. The effects of MPP^+^ were not mediated by D1/D5- and D2-like receptor activation. Inhibition of the dopamine transporters did not prevent but increased the depression of excitatory post-synaptic field potentials. In the search for a possible mechanism, we observed that MPP^+^ reduced the appearance of polyspikes in population spikes recorded in str. pyramidale and increased the frequency of miniature inhibitory post-synaptic currents. The acute effect of MPP^+^ on synaptic transmission was attenuated by co-application of a GABA_A_ receptor antagonist. Taking these data together, we suggest that MPP^+^ affects hippocampal synaptic transmission by enhancing some aspects of the hippocampal GABAergic system.

## Introduction

Parkinson’s disease (PD) is characterized by movement-related motor dysfunctions (e.g., tremor, akinesia, and rigidity; Rodriguez-Oroz et al., 2009). In addition, some patients with PD show an impairment of cognitive functions (Whittington et al., 2006; Lee et al., 2010; Weiermann et al., 2010) and have mild cognitive impairment (MCI; Fernandez et al., 2005; Goldman and Litvan, 2011) or dementia (Emre et al., 2007; Aarsland and Kurz, 2010) including impairments of declarative memory (Bondi and Kaszniak, 1991).

Whereas the genesis of sporadic PD and PD with dementia is still under debate, a correlation between PD cases and the extent of the use of herbicides and pesticides has been described (Liou et al., 1997; Stephenson, 2000; Sun et al., 2007; Tanner et al., 2011). In animal models, the herbicide paraquat causes death of dopaminergic neurons within the substantia nigra (SN), ventral tegmental area (VTA) and as well as the degeneration of the dopaminergic fibers within the striatum (Ren et al., 2009) – a hallmark of PD onset.

Compounds with a structure similar to the herbicide paraquat, have been reported to induce severe motor dysfunction that closely resembles an advanced stage of PD (Przedborski et al., 2001; Dauer and Przedborski, 2003). One such compound is the neurotoxin 1-methyl 4-phenyl 1,2,3,6-tetrahydropyridine (MPTP). Systemic administration of MPTP in primates and mice causes motor dysfunctions, as well as cognitive deficits that correlate with depletion of dopamine in the midbrain (Bove et al., 2005) and hippocampus (Zhu et al., 2011) within several days after its administration. In addition, dopaminergic denervation was shown to potentiate GABAergic inhibition in the mouse neostriatum (Schlosser et al., 1996). It is a common view that MPTP has to be metabolized to 1-methyl-4-phenylpyridinium ion (MPP^+^) via monoamine oxidase in glia cells and that MPP^+^ in turn is transported into dopaminergic neurons via the dopamine transporter (DAT). Since the expression of monoamine oxidase in glia cells is not restricted to the SN or VTA a metabolism of MPTP can take place in other brain areas such as the hippocampus.

Cognitive abilities, such as formation of declarative memory rely partially on the hippocampus (Manns and Eichenbaum, 2006; Morris, 2006) and are known to be modulated by the dopaminergic system. Interference with hippocampal neurotransmission (Bliss and Collingridge, 1993; Morris, 2006) could therefore represent a putative mechanism for altering memory performance in PD patients.

In addition, it is known that a major mesolimbic projection toward the CA1 area of the hippocampus contains dopaminergic fibers originating from the VTA, SN, and pars compacta (Gasbarri et al., 1994, 1997), and that these hippocampal dopaminergic fibers contain the whole machinery required for dopamine synthesis and recycling, including DAT (Lisman and Grace, 2005). However, the putative effects of MPP^+^ on their afferents and subsequently on hippocampal synaptic transmission are not well understood (Galvan et al., 1987). We focused on the acute effects of MPP^+^ on hippocampal excitatory synaptic transmission, to differentiate from the chronic effects of MPTP and MPP^+^.

Here, we looked into the effects of MPP^+^ on synaptic transmission at hippocampal Schaffer collateral (SC)-CA1 synapses using recordings of field excitatory post-synaptic potentials (fEPSPs), polyspikes and miniature inhibitory post-synaptic currents (mIPSCs). The data suggest that MPP^+^ dose-dependently affects synaptic transmission in acute hippocampal slices of C57BL/6 mice via modulation of some aspects of the GABAergic system.

## Materials and Methods

### Animals

Young adult male C57BL/6 mice (8 weeks) were supplied from the animal center of the Chinese Academy of Sciences (CAS, Shanghai, China) and maintained in accordance with the established standards of animal care and procedures of the Institutes of Brain Science and State Key Laboratory of Medical Neurobiology of Fudan University, Shanghai, China. Efforts were made to minimize the number of animals used. Animals had free access to food and water.

In addition, all studies and individual protocols were conducted in accordance with the GSK Policy on the Care, Welfare and Treatment of Laboratory Animals and were reviewed by the Institutional Animal Care and Use Committee either at GSK or by the ethical review process at the Institutes of Brain Science and State Key Laboratory of Medical Neurobiology of Fudan University, Shanghai, China.

### Electrophysiology

#### Hippocampal Slice Preparation

Hippocampal slices were prepared as described previously (Leutgeb et al., 2003; Cai et al., 2010; Zhu et al., 2011, 2012). Briefly, after anesthesia with isoflurane, brains were removed and immersed in pre-carbogenated (95% O_2_/5% CO_2_) ice-cold ACSF (composition in mM: 119 NaCl, 2.5 KCl, 2.5 CaCl_2_⋅2H_2_O, 1.3 MgCl_2_⋅7H_2_O, 1 NaH_2_PO_4_, 11 glucose, 26.2 NaHCO_3_, pH 7.4). A piece of the entorhinal cortex was sliced off and the two hemispheres glued with the midline surface on the slicing platform of the sectioning system. Transverse hippocampal slices (350 μm) were cut and maintained in a submerged-incubation chamber for at least 1 h at room temperature (25°C) and then transferred to a submerged type recording chamber system and further incubated for at least 30 min at 32°C under constant perfusion (4 ml/min) with carbogenated ACSF.

#### Field Potential Recording

Field excitatory post-synaptic potentials were recorded in the stratum radiatum (str. rad.) of the hippocampal CA1 area via borosilicate micropipettes filled with ACSF. Bipolar stimulation electrodes were used to stimulate SC fibers within the str. rad. every minute. Recorded field potentials were amplified by StAmp (Scientifica, UK) and then digitized at a sample frequency of 10 kHz and filtered (1 kHz low-pass, 1 Hz high-pass). The stimulation strength was adjusted to ∼40–50% of the maximum fEPSP-slope value. Paired-pulse facilitation (PPF) of fEPSPs was recorded at a 50 ms inter-stimuli interval.

#### Polyspike Recording

Repeated afferent stimulation of SCs for 30 s at 1 Hz evokes multiple population spikes (PSs) in the CA1 region due to the reduction of GABAergic transmission (Luthi et al., 1997; Saghatelyan et al., 2004). SCs were stimulated with a bipolar platinum electrode placed in the str. rad. at a position ∼400 μm from the recording electrode. ACSF-filled glass pipettes had a resistance of about 2 MOhm and were placed in the str. pyramidale. The mono-phasic stimulation pulse width was 0.2 ms and the stimulation strength was adjusted to evoke a PS of maximal amplitude. The CA3 region was sliced off from the CA1 region to avoid bursts originating from recurrent excitation of the CA3 region. The areas of the first and following PSs were measured and compared to the area of the first spike. To this end a line was drawn manually from the positive peak after the first spike to the recovery of the last detectable negative deflection. In **Figures 4C,D** such lines are indicated with dotted lines. The upper limit of the first spike was defined from the onset of the negative deflection to the first positive maximum.

#### Whole-Cell Voltage-Clamp Recording

Miniature inhibitory post-synaptic currents of CA1 neurons were recorded using 3–5 MOhm pipettes filled with a solution containing (in mM): Cs-gluconate 117, NaCl 2.8, EGTA-acid 0.4, HEPES 20, tetraethylammonium chloride 5, ATP Mg 2, GTP Na_2_ 0.2, glucose 10, pH 7.25. Tetrodotoxin (TTX; 1 μM) and CNQX (25 μM) were added to the ACSF to prevent action potential driven transmitter release and excitatory transmission. mIPSCs were recorded by a Multiclamp 700B amplifier, digitalized with Digidata 1440 and acquired by Clampex 10.2 software (Molecular Devices, Silicon Valley, CA, USA). Membrane potentials were held at -70 mV and currents were processed through 1 kHz low-pass and 0.1 Hz high-pass filters. mIPSC amplitude and frequency were analyzed using MiniAnalysis 6 (Synaptosoft Inc., Fort Lee, NJ, USA). mIPSCs were collected for intervals of three min before (baseline), and 10 and 30 min after MPP^+^ or vehicle application. Statistical comparison of data was performed using the Student’s or non-parametric tests for unpaired or paired samples.

### Statistical Analysis

Maximal fEPSP-slopes were determined and expressed in percentages as mean ± standard error of mean (SEM). The values for different experimental conditions were compared using *t*-test or non-parametric tests (Mann–Whitney *U*-test or Wilcoxon signed-ranks test; SPSS). A *p* < 0.05 was considered to indicate a statistically significant difference between two groups. Brackets are used to indicate the range of significant difference between groups for the corresponding time points (*p* < 0.05). Drug experiments were interleaved with drug-free controls.

## Results

### MPP^+^ Modulates Excitatory Synaptic Transmission of Schaffer Collateral-CA1 Synapses

**FIGURE 1:**
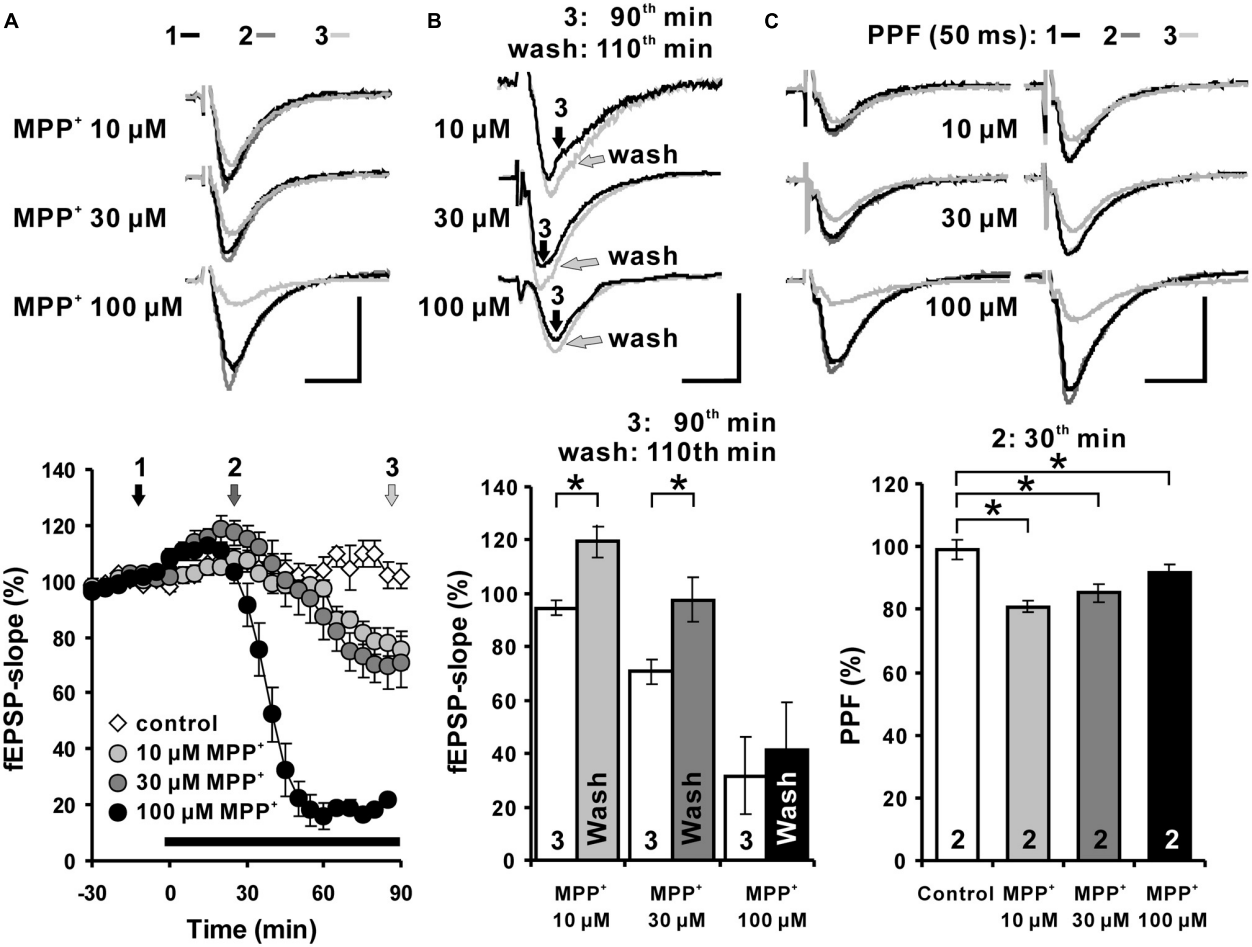
**Acute MPP^+^ application alters excitatory synaptic transmission of Schaffer collateral-CA1 synapses. (A)** MPP^+^ modulates hippocampal synaptic transmission in a concentration-dependent manner. In the CA1 region, application of 10, 30, and 100 μM MPP^+^ caused a concentration dependent fEPSP enhancement followed by a fEPSP depression. The MPP^+^ application started immediately after the sixth baseline recording (-5 min), however, the values, taken 5 min later, have been indicated in the figure as the time point 0, the onset of the MPP^+^ effect. Inserts show sample traces of fEPSPs corresponding to the time points indicated with 1, 2, and 3 in the graphs below. **(B)** fEPSPs recovered partially within 20 min after MPP^+^ wash-out. The white bars show the averaged fEPSP slope values after 90 min of MPP^+^ bath-application and the gray bars the values 20 min after MPP^+^ wash-out. 10 and 30 μM MPP^+^ bath-application caused a depression of fEPSPs, which recovered within 20 min after MPP^+^ wash-out in comparison to the fEPSP slope values following 90 min of MPP application. **(C)** Paired-pulse facilitation (PPF: 50 ms) of fEPSPs is reduced by MPP^+^ within 30 min of MPP^+^ application. The PPF was reduced significantly, however, to a similar degree at all tested MPP^+^ concentrations. Corresponding fEPSP traces are depicted above diagrams. Horizontal scale bars indicate 10 ms and the vertical one 0.5 mV. The black horizontal bar shows the MPP^+^ application period. Small numbers inside the bars indicate the time point of the measurement. Brackets enclose all time points where the group values are significant different from each other (^∗^*p* < 0.05). The compared groups are indicated with their corresponding symbols used in the line graph.

To examine the effects of MPP^+^ on hippocampal synaptic transmission in acute hippocampal slices from C57BL/6 mice, fEPSPs were recorded in the str. rad. of the CA1 region. We observed that in response to the MPP^+^ application an enhancement of fEPSPs was induced within 15 min that was followed by a depression of fEPSPs. We found that 10 μM MPP^+^ decreased the fEPSP slope within 90 min of application to 78.6 ± 4.7%, 30 μM to 69.9 ± 8.3%, and 100 μM to 22.0 ± 1.5%. The averaged fEPSP slope 90 min after vehicle bath-application was 102.3 ± 4.6% (*N* = 2, *n* = 4; **Figure 1A**).

We were interested to learn whether the acute MPP^+^ effect is lasting after wash out.

The degree of recovery after MPP^+^ application was measured 20 min after MPP^+^ wash-out (110th min). The fEPSP slope values 20 min after MPP^+^ wash-out were compared to 90th min values (**Figure 1B**). fEPSPs recovered within 20 min of the wash-out and the fEPSP slope increased from 94.6 ± 2.6% to 119.5 ± 6.1% and from 70.7 ± 4.6% to 97.7 ± 8.1% at the 110th min in the 10 and 30 μM MPP^+^ (*n* = 4) experiments, respectively. The fEPSP recovery after the wash-out of MPP^+^ was significantly different for the 10 and 30 μM MPP^+^ experiments, however, a restoration of fEPSP was not observed after 100 μM MPP^+^ (**Figure 1B**). These results imply a dose-dependent and partially reversible acute modulation of hippocampal synaptic transmission by MPP^+^.

The PPF of 30 and 100 μM MPP^+^ groups decreased to 85.3 ± 4.4% and 91.4 ± 2.6%, and was significantly different from the PPF ratio 99.2 ± 3.2% of the control group. The PPF was reduced significantly, however, to a similar degree at all tested MPP^+^ concentration 30 min after its application (**Figure 1C**).

This shows that the PPF is highly sensitive toward MPP^+^ and already saturated at 10 μM. Whether the MPP^+^ effect on PPF is linked to a reduction of presynaptic vesicle release probability of the available vesicle storage pool as shown for MPTP (Serulle et al., 2007) or to a disturbed interaction of parkin and endocytosis regulating proteins (Trempe et al., 2009) remains to be studied.

### Inhibition of Monoamine Transporters does not Alter MPP^+^ Mediated fEPSP Depression

**FIGURE 2:**
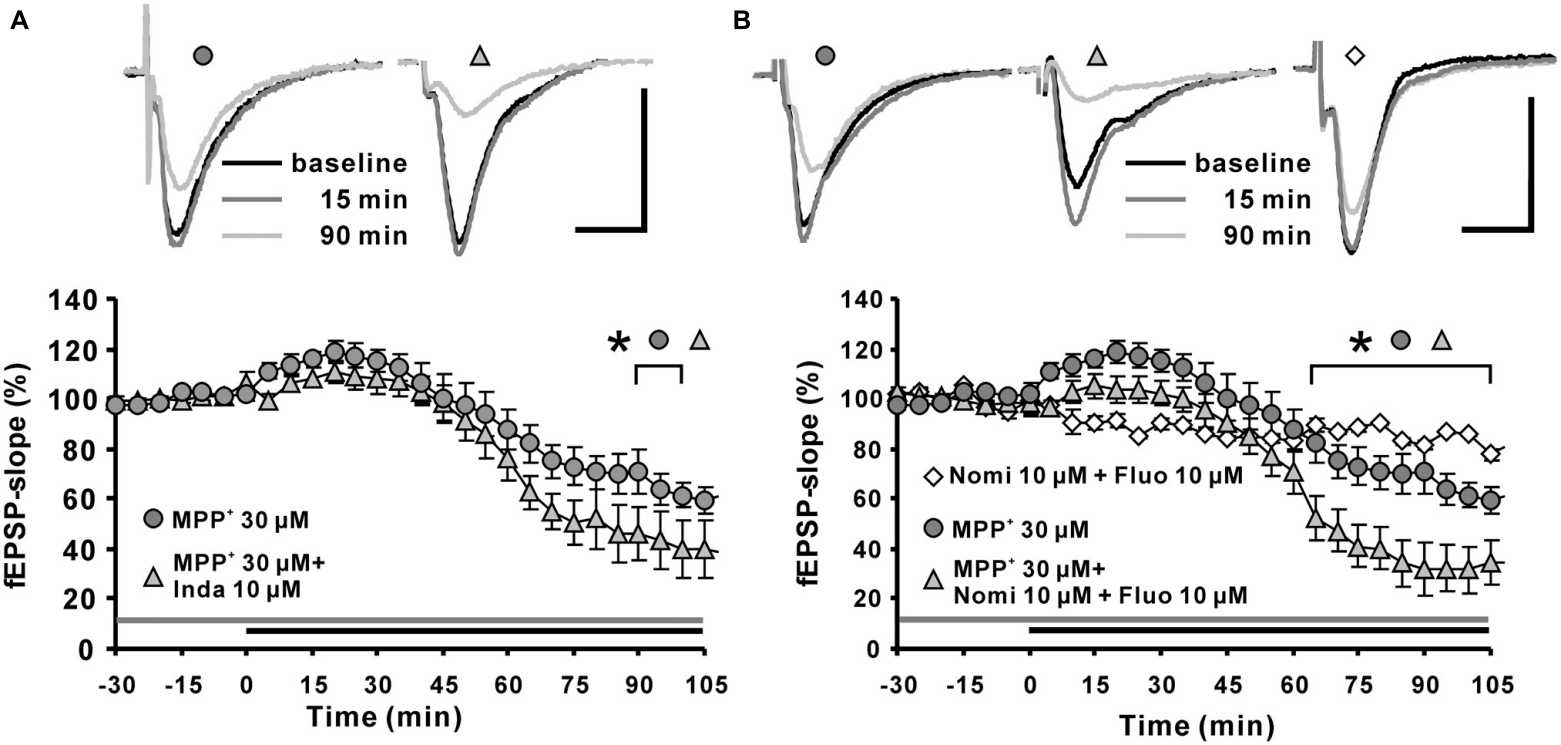
**Effects of monoamine transporter antagonists on MPP^+^ mediated fEPSP depression. (A)** Application of the dopamine transporter (DAT) antagonist indatraline (Inda; 10 μM, light gray filled triangle) 30 min before MPP^+^ application up to the end of recording did not prevent the enhancement of fEPSPs, but increased the fEPSP depression. **(B)** Co-application of nomifensine (Nomi) and fluoxetine (Fluo, light gray triangle) 30 min before MPP^+^ application up to the end of recording facilitated significantly MPP^+^ mediated fEPSP depression in comparison to MPP^+^ application alone (dark gray circles). Co-application of Nomi and Fluo (empty diamonds) did not alter fEPSPs in baseline stability measurements. Baseline sample fEPSP traces and traces for the 15-, and 90-min time points are shown above the graphs. The horizontal scale bar indicates 10 ms and the vertical one 0.5 mV. The application period of MPP^+^ or monoamine transporter antagonists is shown with black or gray horizontal lines, respectively. Brackets enclose all time points where the group values are significant different from each other (^∗^*p* < 0.05). The compared groups are indicated with their corresponding symbols above the brackets.

Toxicological research has shown that MPP^+^ enters dopaminergic afferents through the DAT (Clarke and Reuben, 1995; Kitayama et al., 1998; Storch et al., 2004). In addition, monoamine transporters for norepinephrine and serotonin also have affinities toward MPP^+^ and are responsible to a certain degree for its uptake (Pifl et al., 1996). There are several publications showing that in the hippocampus, such monoamine transporters are expressed in dopaminergic, norepinephrinergic, and serotoninergic afferents (Borgkvist et al., 2012; Tang et al., 2014). In order to examine whether the observed MPP^+^ effect on fEPSP depends on these transporters, we applied the non-selective monoamine transporter inhibitor indatraline, or co-applied this inhibitor with the norepinephrine/DAT inhibitor nomifensine or together with the serotonin transporter inhibitor fluoxetine. After pretreatment with the inhibitors for 30 min to suppress the activity of monoamine transporters in hippocampal slices, we observed that the MPP^+^ mediated depression of fEPSP became enhanced in comparison to control measurements. In particular, co-application of 10 μM indatraline (*N* = 3, *n* = 9) and MPP^+^ decreased fEPSPs to 39.7 ± 11.8% within the 100 min of recording (**Figure 2A**). In comparison to MPP^+^ alone (61.4 ± 4.8%, *N* = 3, *n* = 8) this reduction was significantly different. A similar effect on the MPP^+^ mediated fEPSP decrease was seen after co-application with 10 μM nomifensine and 10 μM fluoxetine (*N* = 3, *n* = 8) that caused a significantly larger MPP^+^ mediated fEPSP depression within 100 min (31.6 ± 9.6%; **Figure 2B**).

Our results indicated that the suppression of monoamine transporters does not attenuate the MPP^+^ effect on hippocampal synaptic transmission, but facilitates the MPP^+^ mediated fEPSP depression. This observation might indicate that a reuptake of MPP^+^ by monoamine afferents is not required for the MPP^+^ mediated fEPSP depression. We could speculate here that inhibition of monoamine transporters might enhance the MPP^+^ availability in the extracellular space.

### MPP^+^ Mediated fEPSP Depression does not Depend on Dopamine Receptors

It has been shown that MPP^+^ administration in the striatum leads to an increase of extracellular dopamine concentration (Obata, 2002; Faro et al., 2009). We could not find data in the literature regarding MPP^+^ induced dopamine release from dopaminergic afferents in the hippocampus; however, it might take place based on the similarities of their dopaminergic pathway. Thus, we further investigated whether MPP^+^ affects hippocampal synaptic transmission by activation of dopamine receptors.

**FIGURE 3:**
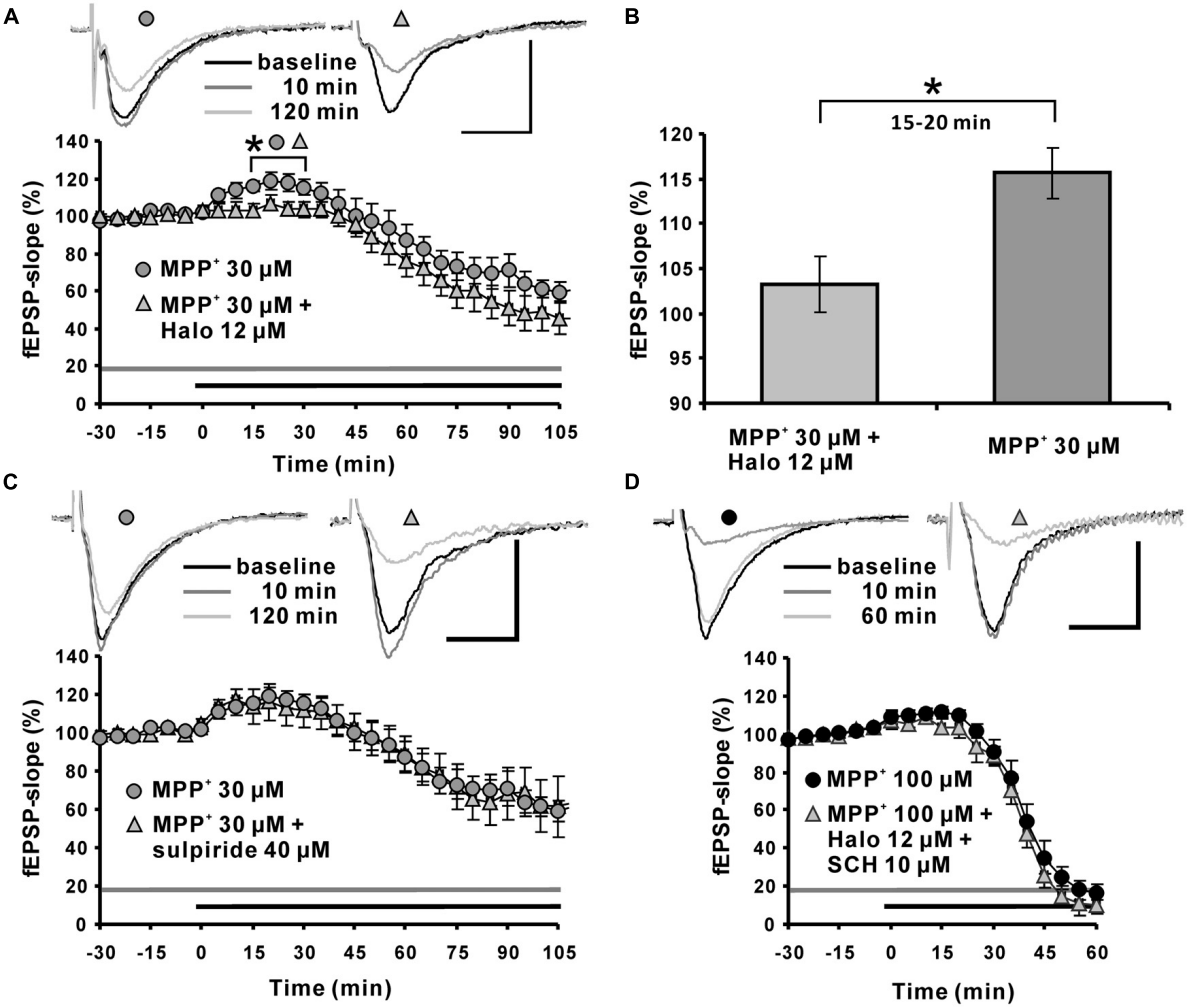
**Role of the dopaminergic system in MPP^+^ mediated fEPSP facilitation and depression. (A)** Application of the D2-like dopamine receptor antagonist haloperidol (Halo; 12 μM, light gray triangles) 30 min before MPP^+^ application up to the end of recording significantly reduced fEPSP-slope facilitation within the first 30 min of MPP^+^ application, but did not alter fEPSP depression induced by MPP^+^ (30 μM, gray circles). Corresponding sample traces are shown above the graphs for time points indicated by black (baseline), dark gray (10 min) and light gray (120 min) lines. **(B)** Bar graph emphasizes the presentation of the Halo effect on 30 μM MPP^+^-mediated fEPSP-slope facilitation for the time point 30 min. **(C)** Application of the D2 receptor antagonist sulpiride (40 μM, light gray triangles) did not alter MPP^+^-mediated fEPSP alterations (dark gray circles). **(D)** Co-application of Halo and D1/D5-like dopamine receptor antagonists (light gray triangle) did not affect the 100 μM MPP^+^-mediated fEPSP-facilitation and depression (black circles). Sample traces are shown above both graphs for each time point as indicated. The horizontal scale bar represents 10 ms and the vertical one 0.5 mV. The application period of MPP^+^ or other compounds is shown with black or gray horizontal lines, respectively. Brackets and asterisks indicate the significant difference between groups (^∗^*p* < 0.05). The compared groups are indicated with their corresponding symbols used in the line graph.

Application of the dopamine D2-like receptor antagonist haloperidol attenuated the MPP^+^ induced fEPSP-slope enhancement within the first 15∼20 min to 103.3 ± 3.1% (*N* = 5, *n* = 7) compared with the 115.6 ± 2.8% in the MPP^+^ alone group (*N* = 3, *n* = 8). However, haloperidol did not alter the MPP^+^ induced fEPSP depression within 120 min of recording (**Figures 3A,B**).

The dopamine D2-like receptor antagonist sulpiride 40 μM (*N* = 2, *n* = 7) did not affect the 30 μM MPP^+^ induced enhancement of synaptic transmission, and did not modulate the corresponding fEPSP depression (20 min: 113.8 ± 9.3%; **Figure 3C**).

At a concentration of 100 μM MPP^+^, 12 μM haloperidol co-applied with dopamine D1-like receptor antagonist SCH23390 10 μM (*N* = 2, *n* = 4) did not antagonize the enhancement of fEPSPs and did not alter the MPP^+^ mediated fEPSP depression (**Figure 3D**).

### Attenuation of Polyspike Appearance Indicates Modulation of the Inhibitory System by MPP^+^

Previous experiments suggested that the MPP^+^ mediated fEPSP modulation does not depend on monoamine transporters and D1/D5- and D2-like dopamine receptors. Since it is known that alteration of the GABAergic system can influence the efficiency of synaptic transmission (Ault and Nadler, 1982; Davies and Collingridge, 1996), we conducted experiments to study the involvement of this inhibitory system in MPP^+^ mediated alteration of excitatory synaptic transmission.

**FIGURE 4:**
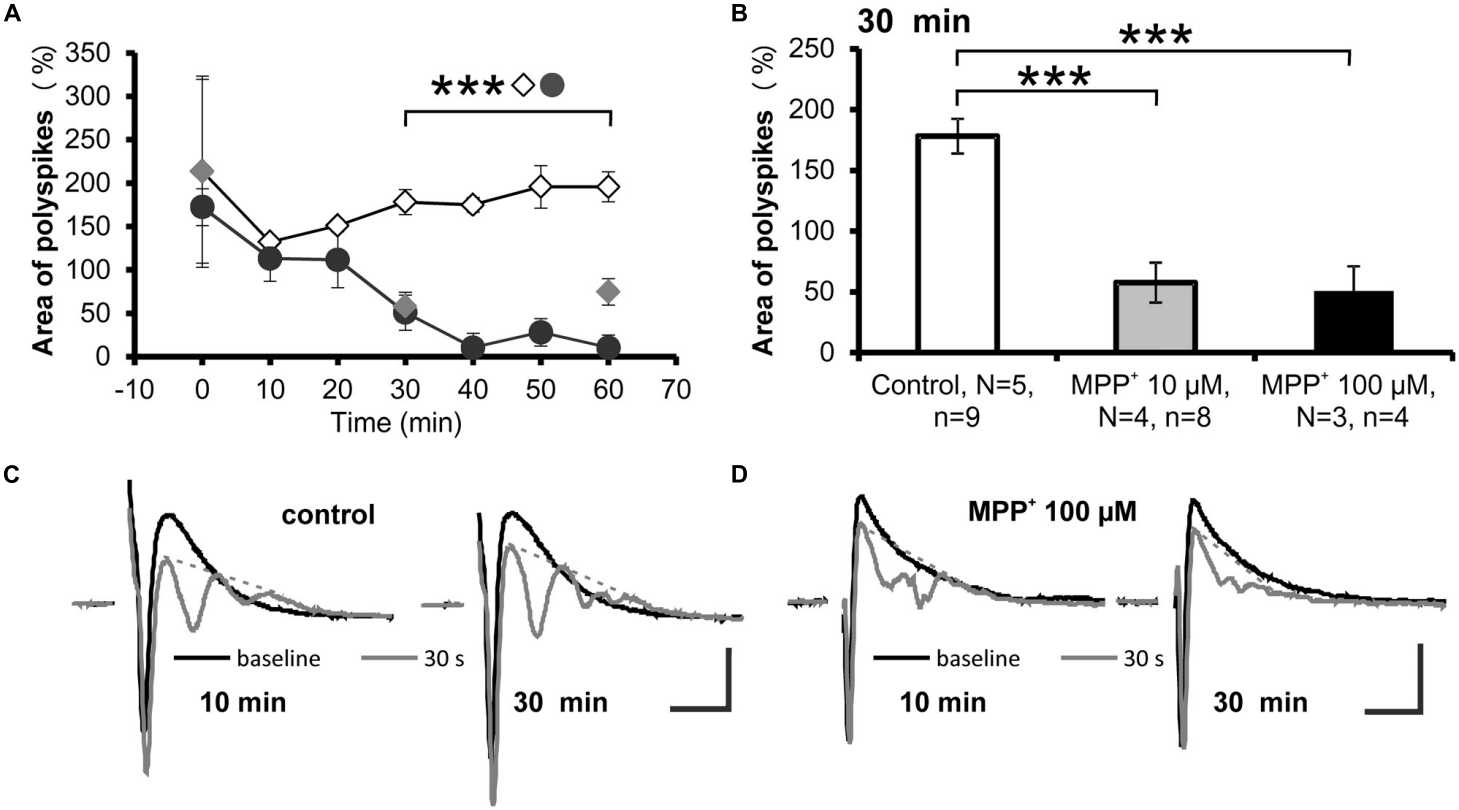
**Reduced polyspike appearance after MPP^+^. (A)** The time course of low-frequency induced polyspike appearance measured every 10 min in experiments with MPP^+^ (10 μM MPP^+^: gray filled circles; 100 μM MPP^+^: black filled circles) and without MPP^+^ (empty diamonds) is shown. ^∗∗∗^*p* < 0.001 between control and 100 μM MPP^+^. **(B)** The bar graph illustrates averaged values of the normalized polyspike area 30 min after start of recording for the control and MPP^+^ experiments. ^∗∗∗^*p* < 0.001 between control and MPP^+^ experiments. Sample traces of the first (black line) and the last (gray line) population spike of the 30 stimuli, 1 Hz train are presented for **(C)** control and **(D)** MPP^+^ experiments. Dashed gray lines indicate extrapolated baselines utilized for polyspike area calculation. Scale bars: 1 mV and 10 ms.

A reduction of the strength of GABAergic inhibition can be evoked by repetitive low-frequency stimulations, which cause the appearance of additional PSs, referred to as “polyspikes” (Saghatelyan et al., 2004). The effect of MPP^+^ on the appearance of polyspikes was analyzed by measuring the area of the first and second PS followed by normalization of the polyspike area to the area of the first spike (**Figures 4A,C,D**). We observed that after 30 min of 10 or 100 μM MPP^+^ application, the normalized area of the second spike was 57.5 ± 16.5% (*N* = 4, *n* = 8) and 50.6 ± 20.4% (*N* = 3, *n* = 4). The value for the normalized area before MPP^+^ application was 178.1 ± 14.4% (*N* = 5, *n* = 9; **Figures 4B,D**). As shown in **Figures 4A,B** these values are significantly different and demonstrate that MPP^+^ increases the strength of the GABAergic system.

### MPP^+^ Affects the Frequency of Miniature Inhibitory Post-synaptic Currents (mIPSCs)

**FIGURE 5:**
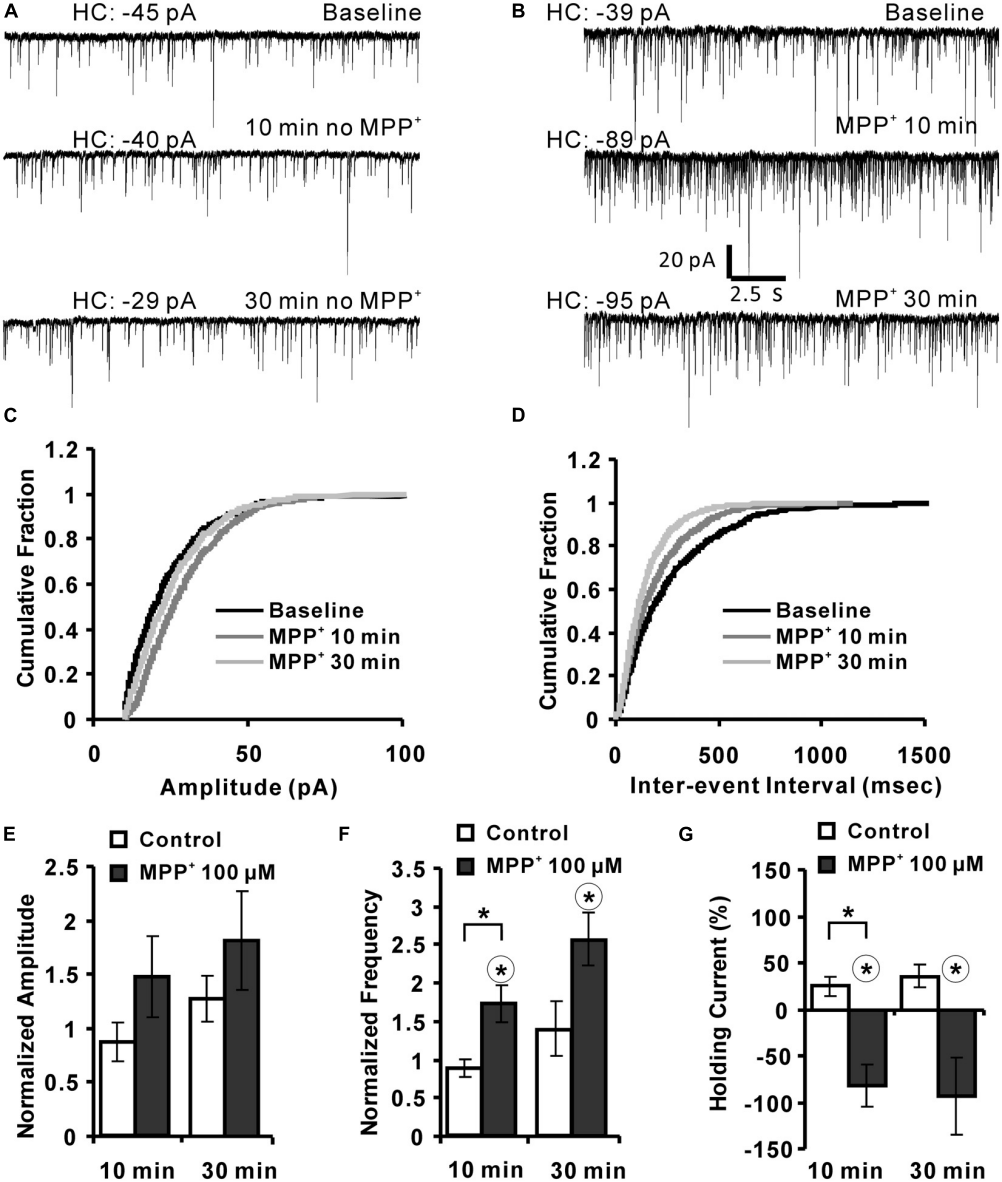
**MPP^+^ alters mIPSC frequency. (A)** Sample traces of mIPSCs before and after vehicle application are shown for the indicated time points. HC values are indicated in pA. **(B)** Whole-cell voltage-clamp recordings are presented for the time points before (baseline), and after 10 and 30 min MPP^+^ wash-in. The diagrams depict the cumulative fractions of mIPSC **(C)** amplitudes and inter-event intervals **(D)** for an individual experiment before (baseline) and following 10 and 30 min of MPP^+^ wash-in. **(E)** Bar graph summarizes normalized mIPSC amplitudes recorded in experiments with and with-out (control) MPP^+^ application. Data are presented for the 10- and 30-min time points of vehicle or MPP^+^ wash-in. mIPSC amplitudes were normalized to the respective values of before the vehicle or MPP^+^ application. **(F)** Bar diagram summarizes normalized mIPSC frequencies. **(G)** The bar graph shows the values of HC normalized to their HC baseline values (first 3 min). In graph **(F,G)** the range of the significant difference between control and MPP^+^ per time point is indicated with brackets and asterisks (*p* < 0.05; Mann–Whitney test). Asterisks in a circle indicate a significant difference of the 10 and 30 min values to their own baseline values before drug application (Wilcoxon paired signed-ranks test).

The previous experiments gave some hints that MPP*^+^* might interfere with synaptic transmission by modulation of an inhibitory system. To further expand this hypothesis, we analyzed the effect of MPP*^+^* on mIPSCs using whole-cell voltage clamp of CA1 neurons. We found that 100 μM MPP^+^ increased the number of mIPSC events to a significant extent in comparison to MPP^+^ free experiments (**Figures 5A,B**). The mIPSC frequency increased to 171.6 ± 24.4% of baseline after 10 min MPP^+^ treatment, which was significantly higher than the 87.8 ± 11.2% of the control experiments (**Figures 5D,F**). In contrast, the amplitude of the mIPSCs was not significantly altered (**Figures 5C,E**). More specifically, after 10 min of 100 μM MPP^+^ (*N* = 6, *n* = 6) the mIPSC amplitude increased to 150.0 ± 37.1% of baseline values, whereas in control measurements the amplitude was 86.9 ± 17.5% (*N* = 2, *n* = 4). 30 min after 100 μM MPP^+^ the mIPSC amplitude increased further to 180.9 ± 45.6% of baseline values (*N* = 4, *n* = 6), whereas in control measurements the amplitude was 127.3 ± 21.4% (**Figure 5E**). The frequency after 100 μM MPP^+^ at 30 min was 255.6 ± 34.7% compared to 138.7 ± 36.0% in control group (**Figure 5F**).

The holding current (HC) at 10 and 30 min was normalized to the baseline holding (B) current [(HC_t_-HC_B_)/HC_B_^∗^100%]. MPP^+^ increased the HC by about 100% within 10 and 30 min. This increase was significantly different to the 10 min value of the control group (*N* = 2, *n* = 4). The HC after 10 and 30 min MPP^+^ application was also significantly different to their baseline values (**Figure 5G**).

### Role of GABA_ergic_ Receptors in MPP^+^-Induced Depression of Synaptic Transmission

**FIGURE 6:**
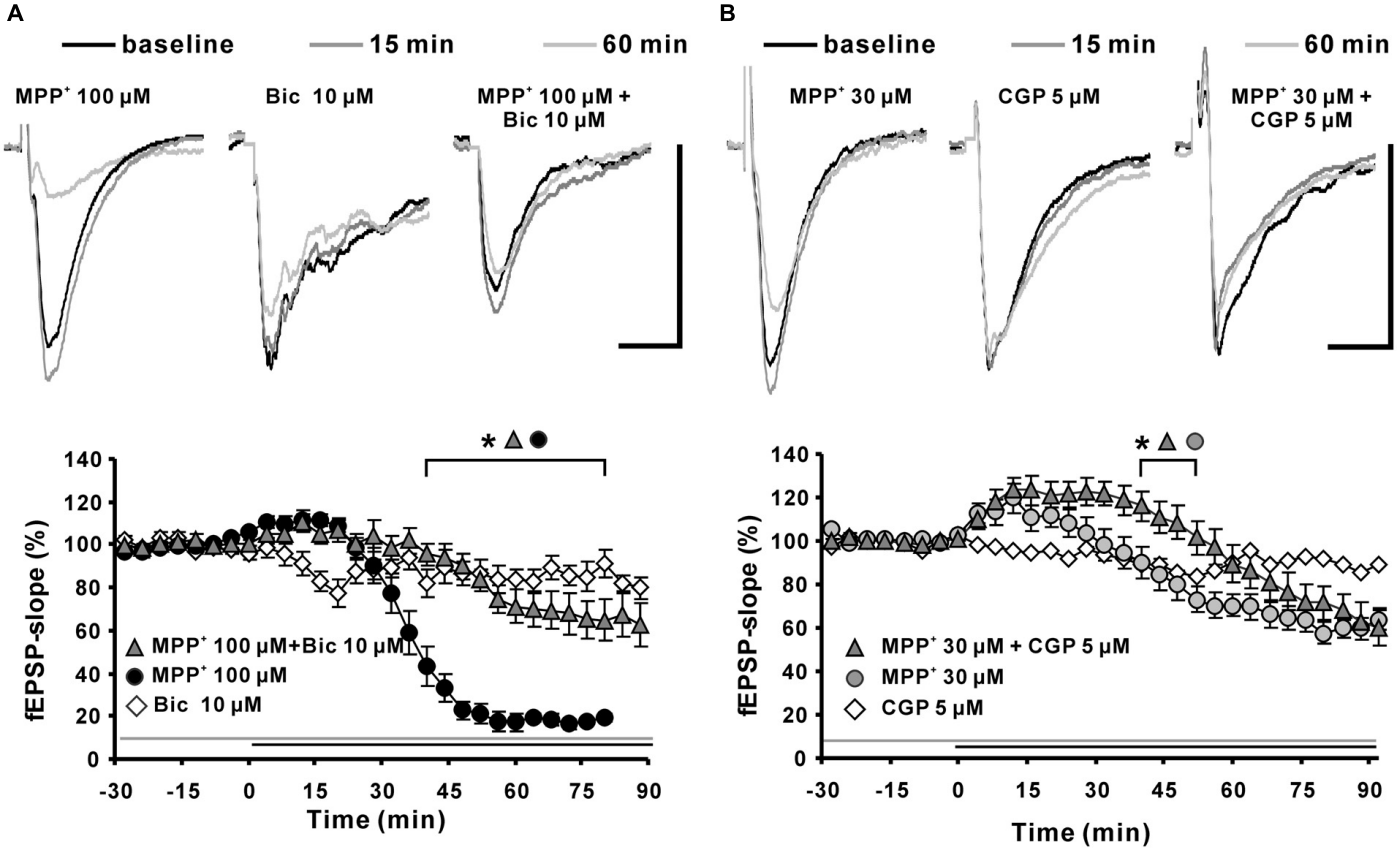
**Effects of GABA_A_ and GABA_B_ receptor antagonists on MPP^+^-mediated fEPSP depression. (A)** Application of the GABA_A_ receptor antagonist bicuculline (Bic 10 μM, light gray filled triangles) 30 min before MPP^+^ 100 μM application up to the end of the recording significantly reduced the fEPSP-slope depression significantly in comparison to MPP^+^ 100 μM application only (black circles). Bicuculline did not alter the fEPSP’s baseline stability. **(B)** Application of the GABA_B_ receptor antagonist CGP52432 (CGP) prolonged the MPP^+^ mediated fEPSP enhancement but did not alter the degree of fEPSP depression. Baseline fEPSP sample traces and traces for the 15- and 60-min time points are shown above the graphs. The horizontal scale bar indicates 10 ms and the vertical one 0.5 mV. The application period of MPP^+^ or GABA receptor antagonists is shown with black or gray horizontal lines, respectively. Brackets enclose the time points with a significant difference between groups (^∗^*p* < 0.05). The compared groups are indicated with their corresponding symbols above the brackets.

To clarify if the MPP^+^ induced fEPSP depression was mediated by activation of GABAergic receptors; we co-applied MPP^+^ together with a GABA_A_ or GABA_B_ receptor antagonist. We observed that the GABA_A_ receptor antagonist bicuculline (10 μM; *N* = 5, *n* = 12) significantly attenuated the decrease of the fEPSP slope value within 45 min of MPP^+^ (**Figure 6A**). The normalized fEPSP slope value at the 90th min was, with 64.6 ± 9.8%, significantly larger than the 19.6 ± 2.0% of the 100 μM MPP^+^ group (*N* = 4, *n* = 8). However, the GABA_A_ receptor antagonist did not prevent the initial fEPSP enhancement by MPP^+^. Application of the GABA_B_ receptor specific antagonist CGP52432 (CGP) did not prevent fEPSP depression, but amplified and prolonged the initial fEPSP enhancement induced by 30 μM MPP^+^ (**Figure 6B**).

Thus, we suggest that bicuculline-sensitive GABA_A_ receptors play a key role in the MPP^+^ induced fEPSP depression and that an inhibitory effect of MPP^+^ on GABA_B_ receptors might be the cause for the initial fEPSP enhancement.

## Discussion

The initial design of the experiments was based on the assumption that MPP^+^ might affect hippocampal synaptic transmission of the dopaminergic system by modulation of dopamine release from nerve terminals and/or effects at dopamine receptors. This assumption was based on data showing that MPP^+^ is actively transported into dopaminergic neurons of the SN by the dopamine transporter complex (DAT) reducing ATP production and loss of mitochondrial membrane potential leading to the damage of the mitochondrial respiratory chain complex. Since hippocampal dopaminergic afferents also contain DAT (Lisman and Grace, 2005) it was reasonable to presume that MPP^+^ induces axonopathy of hippocampal dopaminergic afferents by entering the fibers through DAT. However, in our experiments, none of the tested monoamine transporter inhibitors attenuated the MPP^+^ effect on fEPSPs, but on the contrary, facilitated MPP^+^ induced fEPSP depression. Since application of the inhibitors alone did not affect fEPSPs in a similar manner to MPP^+^, we can exclude interference of MPP^+^ with monoamine transporters as a cause of MPP^+^ induced fEPSP depression.

MPP^+^ is known to induce dopamine release in the striatum by modulation of voltage-dependent calcium channels and reversal of DAT in striatal neurons (Inazu et al., 2001). In addition, in other brain regions with dopaminergic neurons increases of extracellular dopamine in response to the acute MPP^+^ application have been shown (Faro et al., 2009). As mentioned before, hippocampal dopaminergic afferents express the majority of transporters and channels similar to the soma, thus it was assumed that an enhanced dopamine release might contribute to the described MPP^+^ effects. In addition, dopamine D1/D5- and D2-like receptors are expressed on pre- and post-synaptic compartments of hippocampal excitatory neurons (Goldsmith and Joyce, 1994). In contrast to this assumption we did not notice significant effects of D1/D5- and D2-like receptor antagonists on MPP^+^ mediated fEPSP depression. Only the initial enhancement of the fEPSPs was antagonized by blocking D2-like dopamine receptors using the antagonist haloperidol, but not by sulpiride. Since haloperidol is known to antagonize other receptors, such as sigma, 5-HT2 and muscarinic receptors, it might be prudent to suggest that D2-like receptors are not involved in MPP^+^ mediate effects on fEPSPs. In addition, co-application of D2-like and D1/D5-like dopamine receptor antagonists did not alter the dynamic of fEPSP modulation by 100 μM MPP^+^.

Since the present experiments indicated that the hippocampal dopaminergic system is not required in the MPP^+^ induced fEPSP modulation we analyzed whether MPP^+^ causes alteration of the hippocampal inhibitory system. The GABAergic system is involved in regulation of synaptic transmission by activation of GABAergic autoreceptors localized on presynaptic compartments of inhibitory and excitatory neurons (Ault and Nadler, 1982; Davies and Collingridge, 1996). These autoreceptors have been shown to regulate the homeostatic activity of the GABAergic system by feedback inhibition (Axmacher and Draguhn, 2004) and are able to induce post-synaptic shunt effects (Draguhn et al., 2008) by retardation and suppression of the presynaptic vesicle release in excitatory nerve terminals. The down regulation of excitatory vesicle release has been shown for the GABA_B_ receptor agonist baclofen, which activates autoreceptors to enhance feed-forward inhibition and reduces synaptic transmission in CA1 neurons (Davies and Collingridge, 1996). In addition, a study of Ault and Nadler (1982) indicated that 20 μM (±) baclofen can reduce the fEPSP to 20 ± 7% of baseline in the hippocampus SC-CA1 pathway, a level of fEPSP depression similar to our observation. Baclofen is also known for its presynaptic effects in CA1 neurons, for reducing the mEPSC frequency without modulation of mEPSC amplitude. This effect is thought to be mediated by direct binding of baclofen to GABA_B_ receptors and consequently does not alter mIPSC frequency and amplitude (Scanziani et al., 1992).

One way to analyze the strength of the inhibitory circuits in the hippocampus is based on the appearances of polyspikes in response to repeated stimulations and loss of inhibition (Saghatelyan et al., 2004). We found that MPP^+^ attenuates the appearance of polyspikes, which indicates that MPP^+^ enhances some aspects of the GABAergic system. In a further effort to characterize the effects of MPP^+^ on the GABAergic system the amplitude and frequency of mIPSCs were studied. Here MPP^+^ increased the mIPSC frequency without significant alteration of their amplitude. Application of a GABA_B_ receptor antagonist did not prevent MPP^+^ mediated fEPSP depression, but increased the MPP^+^ induced fEPSP enhancement. Thus, the involvement of autoreceptors, mainly mediated by GABA_B_ receptor activation, seems not to be responsible for the MPP^+^ induced fEPSP depression.

However, our experiments with MPP^+^ indicated an alteration of the GABAergic system by other mechanisms, because MPP^+^ enhanced mIPSC frequency and the MPP^+^ induced fEPSP depression was only attenuated by a GABA_A_ receptor antagonist. That enhanced activity of GABA_A_ receptors is able to alter neuronal activity was shown, for instance, for ethanol, which increases tonic inhibition through activation of GABA_A_ receptors, leading to a reduction of fEPSPs in the DG region (Wei et al., 2004). In addition, an increased extracellular GABA concentration was found to enhance tonic inhibition of neuronal circuits with an overall impact on the excitatory activity of neurons (Caraiscos et al., 2004). One of the differences between GABA_B_ and GABA_A_ receptors is that they are prone to desensitization, which might contribute to the low impact of GABA_B_ receptors in tonic inhibition (Kanaide et al., 2007). In addition, the GABA_A_ receptors that are involved in tonic inhibition are based on a different subunit composition and therefore distinguishable from GABA_A_ receptors that are participating in phasic inhibition. The GABA_A_ receptors that mediate tonic inhibition require alpha5 subunits and respond to low, ambient GABA concentration and have an extrasynaptic localization (Caraiscos et al., 2004). The involvement of these alpha5 subunits containing GABA_A_ receptors in a learning task was indicated by an enhanced memory performance of alpha5-null mutant mice (Collinson et al., 2002; Martin et al., 2010) and by memory impairments due to an increase in alpha5 GABA_A_ receptor function (Zurek et al., 2014). In addition, ethanol induced impairments in discriminative tasks rely on alpha5 subunit containing GABA_A_ receptors (Platt et al., 2005).

In contrast to the mechanisms of the MPP^+^ induced fEPSP depression, the initial fEPSP increase by MPP^+^ seems to be not mediated by GABA_A_ receptor activation, because bicuculline does not alter the initial phase. GABA_B_ receptor blockage increased the initial phase, thus there might be some complicated interaction of MPP^+^ within the different components of the GABAergic system. It requires supplementary experiments to decipher these putative interactions.

## Conclusion

Bringing these data together, we suggest that MPP^+^ affects hippocampal synaptic transmission by modulating the hippocampal GABAergic system causing thereby an alteration of the efficiency of excitatory synaptic transmission. Whether MPP^+^ affects GABA_A_ receptors directly or activates them by an increase of the ambient GABA concentrations remains to be investigated.

## Conflict of Interest Statement

The authors declare that the research was conducted in the absence of any commercial or financial relationships that could be construed as a potential conflict of interest.
